# Evaluation of the Palatal Features in Relation to Graft Harvesting in the Saudi Population

**DOI:** 10.3390/medicina61010082

**Published:** 2025-01-07

**Authors:** Razan Alaqeely, Sumaiah Ajlan, Faisal Alsanqar, Abdulmahsin Alaqil, Abdulaziz Almansour, Mohammad A. Aldosari

**Affiliations:** 1 Faculty in Periodontics, Department of Periodontics and Community Dentistry, College of Dentistry, King Saud University, Riyadh P.O. Box 11545, Saudi Arabia; 2 Dental Interns at the College of Dentistry, King Saud University, Riyadh P.O. Box 11545, Saudi Arabia; 3 Faculty in Pediatric Dentistry and Orthodontics, College of Dentistry, King Saud University, Riyadh P.O. Box 11545, Saudi Arabia

**Keywords:** palatal anatomy, greater palatine artery, periodontal grafting, palatal rugae, Saudi population

## Abstract

*Background and Objectives:* The palate’s morphological characteristics are of great importance, especially in periodontology, where the palatine tissue represents a source of tissue graft for multiple mucogingival surgeries. This study aimed to estimate the amount of donor tissue available through the average palatal height and average location of the greater palatine artery in the Saudi population according to age and gender. *Materials and Methods:* Digital casts for adult Saudi patients at the age of 18–60 years old with a mean age of 37.76 ± 12.68 years were collected and analyzed using EXOCAD software. The digital casts were evaluated, and measurements of arch width at molars and canines, palatal vault height (PVH), palatal height index (PHI), and extension of the palatal rugae were registered. Finally, the amount available for soft tissue graft harvesting was calculated. *Results*: Overall, 109 maxillary casts for Saudi patients, 52 (47.7%) males and 57 (52.3%) females, were analyzed. The maxillary inter-molar arch width, inter-canine width, and palatal vault height significantly differed between males and females (*p* < 0.05). The mean PHI was 45.51% ± 8.12%, and 27.5% were classified as orthostaphyline, while 72.5% were categorized as hypsistaphyline, with no significant difference between the genders. The mean maximum graft width was 11.45 mm, and the graft width was significantly different between males and females (*p* < 0.005), while the mean maximum graft length was 17.78 mm, and the graft length showed no significant difference. *Conclusions:* The results of this study provide specific clinical guidelines for periodontal procedures by emphasizing the importance of gender-specific anatomical considerations. Data on graft dimensions and palatal measurements will enable the exact planning of soft tissue harvesting to minimize surgical risks and optimize mucogingival surgery outcomes in the Saudi population.

## 1. Introduction

The human palate, an essential part of the cranium, separates the nasal and oral cavities and is divided into two sections: the soft and hard palate. Its bony parts are formed from the fusion of the palatine process of the maxilla and horizontal plates of the palatine bone [1].

The hard palate comprises compact bone, the thickness of which is quite variable and thus represents the most important feature affecting its value in surgical and dental procedures. A study by Aleshkina et al. [2] underlines the importance of the quantitative evaluation of bony palate thickness for the improvement of donor tissue harvesting and complication avoidance during surgeries.

The palate is covered by the masticatory mucosa composed of stratified squamous keratinized epithelium. It has a submucosal region consisting of connective tissue, where other critical anatomic structures are found, including fat tissues and minor salivary glands. The greater palatine artery, a branch of the third part of the maxillary artery, provides blood to the palate. This artery enters the area through the greater palatine foramen and supplies two-thirds of the hard palate by passing anteriorly in a curved manner, close to the alveolar ridge, and becoming smaller and more branched as it proceeds forward [3].

The palate’s morphological and physical characteristics are crucial in several medical and dental fields, including forensic medicine, prosthodontics, orthodontics, oral surgery, and periodontics [4]. In periodontology, the palatine tissue represents a source of tissue grafts for multiple mucogingival surgeries [5], including connective tissue grafting for root coverage and free gingival grafting for gingival augmentation procedures. However, proper knowledge of the palatal anatomy is essential for periodontal practice, as specific anatomical landmarks can limit the amount of tissue that can be harvested [6,7]. For example, the posterior extent of the palatal rugae area is a vital structure, given that harvesting a free gingival graft that includes the palatal rugae tends to re-establish its shape at the newly grafted site, leading to an unaesthetic appearance. Said et al. reported that the palatal rugae often extend beyond the mesial aspect of the maxillary second premolars [8]. The thickness of the bony palate is another important criterion that needs to be evaluated, as it may affect the placement of orthodontic fixation screws. AlEshkena et al. found the greatest bony thickness in the anterior third of the palate and that the bone was significantly thicker for male patients [2].

Another important landmark is the greater palatine artery. Care must be taken to avoid damaging this artery during graft harvesting. Several authors have attempted to evaluate its exact location relative to clinical landmarks to help clinicians identify the safety zones for graft harvesting. Klosek and Rungruang [7] reported that this artery’s location and branching pattern vary significantly between sexes. Similarly, Reiser et al. [9] reported that the artery’s location varies according to the shape of the palatal vault, with the location being 7, 12, and 17 mm from the cementoenamel junction of the maxillary first molars in shallow, moderate, and deep palatal vaults, respectively. This underscores the need for a thorough knowledge and evaluation of palatal vault dimensions in clinical practice. The shape of the palatal vault has been evaluated in other studies, including that by Sergani et al., who recognized that its shape and dimensions varied between sexes and ethnic groups [10]. That is why bone density, thickness, and vault shape are critical factors that should be considered to enhance surgical precision and outcomes.

However, there is a dearth of data on the palatal pattern in the Saudi population, particularly concerning age and sex. Therefore, this study aims to fill this gap by determining the unique average palatal height and average location of the greater palatine artery in the Saudi population. We also estimate the extent of the palatal rugae to evaluate the amount of donor tissue available for grafting procedures. Thus, this study provides insight into the palatal morphology of the Saudi population, representing a significant gap in the literature. Palatal dimensions, the location of the greater palatine artery, and the tissue graft availability regarding gender and age will help clinicians with safe and effective surgical intervention in periodontal procedures. These findings have direct clinical implications because they will allow for adequate surgical interventions with minimum risks and complications and hence with the best outcomes in mucogingival surgeries. Therefore, we hypothesize that significant variations in palatal morphology exist between genders and among different age groups within the Saudi population by influencing the dimensions of donor tissue available for grafting procedures.

## 2. Materials and Methods

### 2.1. Study Population

This cross-sectional study used the cast records of Saudi patients aged 18–70 years who had previously undergone dental treatments at the College of Dentistry, King Saud University. Ethical approval for this study was granted by the Institutional Review Board (IRB) of King Saud University (approval number: E-23-8311; Date: 23 November 2023). The inclusion criteria ensured that only patients with complete dental records and precise digital casts (to guarantee high-quality digital input to the analysis, here digital casts were considered “precise” if a minimum of 0.1 mm resolution of the scan captured the minute anatomical detail, completely mapped out the palatal region covering landmark points such as palatal rugae and gingival margins, and did not include artifacts or noise that might be measurement-critical in those areas) of the maxillary arch were selected for analysis. Records for male and female Saudi patients who had previously undergone dental treatments at the College of Dentistry, King Saud University, were initially screened. Casts for Saudi adult patients aged 18–70 were selected if they had scanned the entire palatal areas involving both sides, and a complete set of posterior teeth was available. The exclusion criteria included the following: (1) patients with a history of bone disease; (2) congenital anomalies or major surgery affecting the palate; (3) history of orthodontic treatment; (4) extraction or congenital absence of more than two posterior teeth per quadrant, excluding the third molars; (5) fixed dental appliances in the upper jaw; (6) severe maxillary posterior teeth malposition; (7) advanced periodontal destruction affecting the maxillary posterior teeth.

### 2.2. Sampling Technique

A stratified proportional sampling method ensured balanced distributions and meaningful comparisons across different life stages. The available records were reviewed for both sexes (male and female) and categorized into adjusted age groups for homogeneity like 18–29 years, 30–44 years, and 45–70 years based on developmental and age-related morphological studies. The 18–29 group represents early adulthood, the 30-44 group reflects middle adulthood, and the 45–70 group represents older adulthood, providing insights into anatomical differences in the Saudi population by being aligned with the prior study by [11].

### 2.3. Data Collection

A total of 109 digital casts were collected from the digital scanning machine (Ceramill Map 400, Amann Girrbach, Mäder, Österreich, Austria), and the dimensions were measured using EXOCAD software version 3.1 (EXOCAD DentalCAD 3.1; EXOCAD GmbH, Darmstadt, Germany). The computer used was Asus (Asos ROG strix G15 G513RM-HQ257, processor: AMD Ryzen 7, ASUSTeK Computer Inc, Taiwan). On each digital cast, the following reference lines were established.

- Mid-palatine line: A line extending posteriorly from the incisive papilla to the end of the palate.
- Estimated location of the greater palatine artery: A line drawn at the junction of the vertical and horizontal parts of the palate [9,12].
- The distal extension of the palatal rugae: the location of the border of the most posterior rugae in relation to the premolar teeth and categorized relative to the second premolar [8]—
  - Mesial to the second premolar.
  - Middle of the second premolar.
  - Distal to the second premolar.

Following the above, the digital cast size was evaluated using the following measurements.

**Figure 1:**
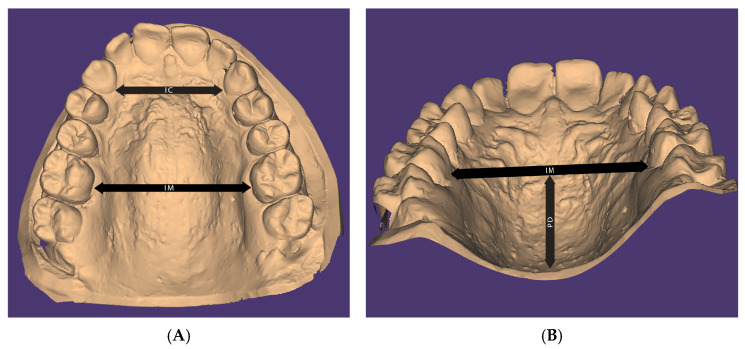
(**A**) Digital cast showing the inter-molar (IM) and inter-canine (IC) arch width. (**B**) Digital cast showing the palatal vault height measurement, which is the palatal depth (PD) at the midpoint of inter-molar (IM) distance.

**Figure 2:**
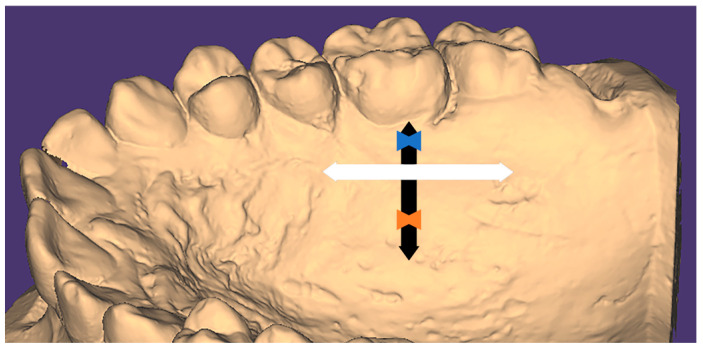
Digital cast showing the graft acquisition, where measurements are taken mesiodistally (white arrow) and apicocoronally (black arrow). Blue and orange arrowheads resemble the subtractions; blue is 2 mm from the gingival margin, and orange is 3 mm from the estimated greater palatine artery.

1. Palatal dimensions
  - Maxillary arch width
    The maxillary arch width at molars and canines (inter-molar [IM] and inter-canine [IC] width): Measurements extending from the gingival margin of the contralateral canines and first molars were taken (Figure 1A).
  - The palatal vault height (PVH): A vertical line was drawn from the midpoint of the IM horizontal line to the mid-palatine raphe (palatal depth PD) [13] (Figure 1B).
  - Palatal height index (PHI): This was measured using the following formula—(Palatal height)/(Palatal width) × 100 [14]. Then, the palate was classified according to the following—
    - Chamestaphyline (low palate) if the values were <27.9%.
    - Orthostaphyline (medium palate) if the values ranged from 28% to 39.9%.
    - Hypsistaphyline (high palate) if the values were >40%.
2. The maximum possible amount of gingival graft acquisition [12]: The graft dimensions, length, and width were calculated according to mesiodistal and apico-coronal extension as follows (Figure 2).
  - Mesiodistal extension (graft length): From the center of the second molar to the margin of the most distal rugae.
  - Apicocoronally (graft width): The distance in millimeters from the deepest part of the palatal cervical margin of the first molar to the estimated location of the greater palatine artery, with a subtraction of 5 mm to allow for a 3 mm safety margin away from the artery and 2 mm away from the gingival margin of the tooth to avoid recession.

### 2.4. Statistical Analyses

The sample size was estimated using G*Power based on alpha error = 0.05, given the effect size of 0.7 and a power of 0.90 (1–β). Therefore, the minimum sample size for the calculation was 90 [15].

Three examiners were involved in data collection, and inter-examiner reliability was evaluated by randomly selecting six different palatal sections evaluated separately by each examiner. Intra-examiner reliability was assessed to ensure the measurements were consistent after 24 h using intra-class correlation (ICC) coefficients.

Data were analyzed using IBM SPSS statistical software version 29 (IBM SPSS Statistics 29; IBM Corp., Armonk, NY, USA). Descriptive statistics were presented as means and standard deviations. The *t*-test and Chi-square test of correlation were used to compare the two groups (male and female), and an analysis of variance was performed to analyze the effect of age on palatal variables, with statistical significance set at *p* < 0.05.

## 3. Results

### 3.1. Intra-Examiner Reliability, with Coefficients

The inter-examiner reliability test showed excellent reliability, where the values ranged from 0.89 to 0.94 using intra-class correlation (ICC). These measurements were repeated after 24 h, but no significant variations were detected among repeated assessments.

Data were recorded using EXOCAD software version 3.1 (EXOCAD Dental CAD 3.1; EXOCAD GmbH, Darmstadt, Germany) for 109 maxillary casts of Saudi patients—52 (47.7%) males and 57 (52.3%) females. The patients’ ages ranged from 18 to 70, with a mean age of 37.76 ± 12.68 years. According to the electronic file system, 90 (82.6%) patients were unaware of any relevant medical condition. The 45–70 age group had the highest number of medical conditions unrelated to the palate (n = 12, 11%).

### 3.2. Palatal Dimension

#### 3.2.1. Maxillary Arch Width

- Inter-molar distance (IM): The mean IM was 34.56 ± 3.8 mm, ranging from 21.49 to 43.45 mm. Male individuals showed a wider distance (36.4 ± 2.8 mm) than female individuals (32.8 ± 3.7 mm), with a significant difference between the sexes (*p* = 0.000) but not between the different age groups.
- Inter-canine (IC) width: The mean IC width was 24.49 ± 2.5 mm in the 19.23–34.11 mm range. Male individuals had a mean width of 25.1 ± 2.1 mm, while female individuals had a mean width of 23.8 ± 2.6 mm, with a significant difference observed (*p* < 0.05).

#### 3.2.2. Palatal Vault Height (PVH)

The PVH ranged between 10.25 and 22.47 mm, averaging 15.61 ± 2.7 mm. Male individuals exhibited a deeper mean PVH (16.7 ± 2.6) compared with female individuals (14.6 ± 2.6 mm)—a difference that was found to be statistically significant (*p* < 0.05). Age did not affect the PVH.

#### 3.2.3. Palatal Height Index (PHI)

**Figure 3:**
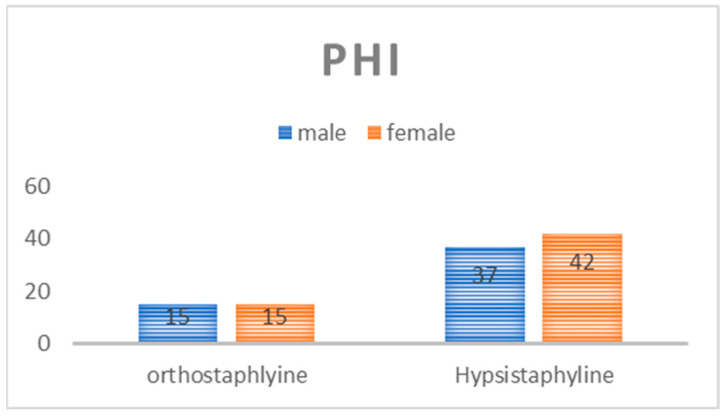
Palatal height index distribution between males and females.

The mean PHI was 45.51% ± 8.12% in the 29.03%–68.7% range. Among the patients, 30 (27.5%) were classified as orthostaphyline (medium palate), while 79 (72.5%) were categorized as hypsistaphyline (high palate). The distribution between sexes is shown in Figure 3. No significant difference was observed between the sexes (*p* > 0.05).

### 3.3. Extension of the Palatal Rugae

A chi-square correlation was performed to identify the differences, which revealed that although there were minor differences regarding the extension of the rugae between the right and left sides of the palate, this was statistically insignificant (*p* > 0.001), indicating symmetrical distribution.

The middle of the second premolar was the most common extension in both genders (Table 1). Age was not correlated with rugae extension on both sides (*p* = 0.6 on the left and *p* = 0.8 on the right).

### 3.4. Estimated Location of the Greater Palatine Artery

The average position of the greater palatine artery from the gingival margin was 16.61 ± 2.3 mm on the right side and 16.29 ± 2.2 mm on the left side, with a statistically significant difference (*p* < 0.001). In males, the position of the greater palatine artery was measured at 17.76 ± 2.2 mm on the right and 17.46 ± 1.9 mm on the left side. In females, the measurements for the right and left sides were 15.56 ± 1.9 mm and 15.23 ± 2 mm, respectively. The difference between males and females was significant (*p* < 0.001).

### 3.5. The Maximum Possible Amount of Gingival Graft Acquisition

- The overall mean maximum graft width was 11.45 mm, with a mean of 11.61 mm and 11.29 mm on the right and left sides, respectively. Male individuals exhibited a mean graft width of 12.7 ± 2.2 mm on the right side and 12.4 ± 1.92 mm on the left side, while female individuals had a mean graft width of 10.5 ± 1.9 mm on the right side and 10.2 ± 2 mm on the left side, with a significant difference (*p* < 0.05).
- The overall mean maximum graft length was 17.78 mm, with a mean of 17.64 mm on the right side and 17.92 mm on the left side. However, no significant difference in graft length was observed between male and female individuals (*p* > 0.05), with measurements of 17.7 ± 3.1 mm and 17.9 ± 2.5 mm for male individuals and 17.5 ± 2.2 mm and 17.8 ± 2.3 mm for female individuals, on the right and left sides, respectively.

In Table 2, the correlation between the right and left sides of the graft is significant (*p* < 0.05), indicating a strong positive correlation. A significant correlation existed between IM and graft length (*p* < 0.05). However, no correlation was observed between the graft width and rugae (*p* > 0.05). A significant correlation was also observed between the PVH, IM, and graft length (*p* < 0.05).

## 4. Discussion

This study aimed to evaluate the palatal dimensions among Saudi populations to predict the amount of donor tissue available for different periodontal grafting procedures with the hypothesis that significant variations in palatal morphology exist between genders and among different age groups within the Saudi population, which influence the dimensions of the donor tissue available for grafting procedures. This study noted significant gender-based differences in most evaluated parameters, so the hypothesis regarding gender-based variations in palatal morphology is accepted. Specifically, the evaluated intermaxillary (IM) and inter-canine (IC) distance as well as palatal vault height (PVH) differed between males and females, with males exhibiting larger readings. This was also consistent with previous research, which indicated the presence of sex-specific anatomical differences in both general and dental arch dimensions, especially where males tend to have larger measurements due to skeletal and dental developmental disparities in response to hormonal factors and genetic predispositions that affect bone and cartilage development [16,17].

For instance, the work of Avci et al. [18] showed that males have larger craniofacial dimensions due to the influence of hormonal and anatomical factors on bone development.

These sex-related differences highlight the importance of incorporating gender-specific anatomical considerations in clinical and dental practices. The noticeable contrast (with a significance of less than 0.05) highlights the importance of considering these differences in real-world healthcare settings, such as orthodontics, maxillofacial and periodontal surgeries, and restorative and prosthetic dentistry, where accuracy in measuring dental arches is vital for planning treatments [19].

On the other hand, insignificant differences were detected among the age groups in previously mentioned parameters, and the hypothesis regarding age-based variations is rejected. This indicates that most palatal measurements remain relatively stable throughout adulthood [10]. Minor age-related changes in craniofacial measurements have also been reported. Moreover, the lack of variation across age groups further reinforces the generalizability to adult populations. It suggests that IM distance is deemed reliable for both male and female patients throughout adulthood [20].

Although the broader anthropometric study emphasized the genetic and hormonal determinants of craniofacial structures [21], the findings were not directly transferable to pediatric or geriatric populations, as these groups experience growth spurts or age-related changes in bone density that affect IM [22]. Therefore, additional studies are suggested to determine the practicability of our results over a wider age span, mainly including younger and older patients. However, those findings should be interpreted cautiously as they may not apply to populations beyond the adult age group. Dental arch width has been shown to change during childhood growth spurts and in life due to age-related dental transformations [23]. Henceforth, conducting research involving individuals of various age ranges would be beneficial in assessing the relevance of these discoveries in children and older adults. Craniofacial structures, including palatal vault height, might experience subtle age-related changes due to factors such as bone remodeling or dental wear [24]. On the other hand, insignificant differences were detected among the age groups in previously mentioned parameters, and the hypothesis regarding age-based variations is rejected. This indicates that most palatal measurements remain relatively stable throughout adulthood.

Interestingly, the palatal height index (PHI) findings revealed that most individuals in this study had a high palate. At the same time, a smaller proportion was categorized as having a medium palate. This is in accordance with previous reports indicating the predominance of high palates in various populations [25]. At the same time, insignificant differences in PHI were observed between the sexes due to genetic and developmental factors [26]. The absence of sex differences in this study indicates that PHI found relatively stable anatomical features across genders, making it a potentially reliable parameter for both male and female patients in clinical practice. At the same time, insignificant PHI among genders highlights the multifaceted interaction between genetic, environmental, and hormonal factors that influences craniofacial development. Even though a study by Fatima et al. [27] reported subtle variations in palatal shape and height between males and females, the present results showed that these differences are not always significant, especially when considering broad population samples [28].

In addition, the rugae extension outcomes revealed a symmetrical distribution between the right and left sides of the palate. Interestingly, this relative bilateral symmetry trait has been noted and utilized in forensic and anthropological research for human identification [29,30], as its unique yet stable patterns provide reliable markers throughout life [30].

It has been further identified that the most common point of rugae extension is located in the middle of the second premolar. This is consistent with existing studies that identified this region as a frequent site of rugae termination in both sexes [31]. This consistency across genders further highlights the stability of rugae as an anatomical feature unaffected by sex differences, which contrasts with other craniofacial structures often influenced by hormonal or developmental factors [32,33].

This research further revealed that age was not correlated with rugae extension on either side of the palate, reinforcing the idea that palatal rugae remain unchanged after early adulthood. This firmness makes rugae a valuable tool in forensic identification and dental practice for treatment planning [31]. It further indicates that rugae extension is generalizable across adult populations with minimal influence from external factors such as aging or wear [34].

Correspondingly, the GPA’s positional data further provided valuable insights into its anatomical variability and implications for surgical procedures, where the greater palatine artery’s location with notable differences between sexes highlighted the importance of gender-specific considerations in surgical planning. It has therefore been concluded that the artery’s proximity to the gingival tissue must be carefully managed to avoid complications such as excessive bleeding or injury during graft harvesting [35]. In addition, the GPA’s average position was more posterior in males than females, implying that male patients have slightly different surgical considerations. Anatomical studies also reported gender-based differences in the location of critical oral structures [36,37]. That is why accurate mapping of the GPA is vital for minimizing risks and ensuring successful outcomes in gingival graft procedures.

This research also considered the maximum graft length and width and observed significant differences between sexes, with males exhibiting longer grafts and likely having larger oral and gingival tissues [38]. It also studied the stability of graft width across genders and sides. One more outcome revealed a positive correlation between graft length and IM distance, highlighting the role of broader anatomical structures in determining the availability of gingival tissue. A greater IM provides a larger area for grafting, which is particularly relevant in periodontal and reconstructive surgeries [39].

The strong connection between graft length and PVH adds weight to the influence of the overall craniofacial dimensions on graft acquisition. As PVH increases, the potential for obtaining longer grafts increases, which is central for procedures requiring substantial graft volumes [40]. Linking craniofacial morphology with surgical outcomes highlights the need to consider these dimensions in preoperative assessments [41]. Also, a significant correlation between graft length and IM suggests that larger distances facilitate the harvest of longer grafts, which is advantageous in complex surgical cases [42].

Some of the most important benefits of this study include its detailed analysis and presentation of data regarding palatal dimensions, the location of the greater palatine artery, and the extent of the rugae. It thus covers a very important knowledge gap in this particular domain. Such findings are relevant to the direct relationship they present on gender-based differences in graft availability for periodontal procedures; this may lead clinicians to better tailor their approaches during surgeries. Moreover, the EXOCAD software allows them to perform very accurate digital measurements, further demonstrating how significant such advanced imaging tools may be in anatomical studies.

Despite these strengths, this study has a few limitations. First, this institution-based study might have reduced the generalizing capacity for other regions of Saudi Arabia or all ethnic groups. Slight variations in the quality of different digital casts and also the precision of software could produce some measurement errors. Moreover, all parameters assessed in this investigation were related only to anatomical aspects without the evaluation of the functional outcomes of these parameters, such as graft success rates or recovery times. Finally, this study’s cross-sectional design prohibits any temporal conclusions about changes in palatal morphology over time or across different life stages.

This study relies on digital casts from a single dental institution in Saudi Arabia, which may limit the generalizability of the findings to the broader Saudi population or other ethnic groups. In addition, variations in digital cast quality and measurement precision, even with advanced software like EXOCAD, could introduce minor errors or inconsistencies in the data [43]. Thus, to control and decrease these influences, efforts were made to ensure high inter- and intra-examiner reliability through repeated measurements and consistency checks. Also, while adequate for preliminary insights, the sample size does not fully capture the diversity of palatal anatomy across different age and gender groups.

This study recommends future research to expand knowledge on palatal anatomy, particularly in Saudi Arabia. Larger-scale studies should be conducted to determine consistency across different subpopulations and regional variations. Those studies should further evaluate anatomical features such as palatal tissue thickness and type and explore the relationship between palatal anatomical features and demographic factors, such as socioeconomic status, lifestyle, and health conditions. Comparing palatal anatomy across different ethnic groups could contextualize findings globally. Advanced digital imaging and measurement technologies, such as 3D modeling and artificial intelligence, could also be explored for their impact on surgical planning and execution. The cost-effectiveness of personalized grafting techniques based on specific palatal measurements could be explored. The findings could also be integrated into dental education programs to better prepare future practitioners. These research directions could influence the dental field by developing targeted clinical guidelines, innovative technologies, and educational advancements.

## 5. Conclusions

This study concludes with important implications for using gender-specific assessments in clinical practice regarding the Saudi population’s anatomical differences in palatal tissue. First, this research emphasizes the need for accurate anatomical information to improve the efficacy and safety of mucogingival operations. Understanding the intricate fluctuations in palatal vault height and arch dimensions enables more customized and patient-specific methods, eventually enhancing surgical results and patient contentment.

In addition, the findings underline the necessity of developing population-specific clinical guidelines that reflect the unique anatomical characteristics observed in this study. This approach supports more effective periodontal practices and highlights the broader impact of such research on improving healthcare delivery in specific demographic groups. Additionally, integrating advanced digital tools in this study further reinforced the potential for technology to revolutionize dental practice. It suggests a need for continued exploration and incorporation of innovative technologies in clinical and educational settings.

## Figures and Tables

**Table 1:** Posterior extension of palatal rugae between right and left sides between the genders.

	The Right Side of the Rugae (n, %)			*p*-Value	The Left Side of the Rugae (n, %)			*p*-Value
	Mesial	Middle	Distal		Mesial	Middle	Distal	
Male	15 (28.8%)	20 (38.5%)	17 (32.7%)	0.377	15 (28.8%)	24 (46.2%)	13 (25%)	0.279
Female	18 (31.6%)	27 (47.4%)	12 (21.1%)		12 (21.1%)	35 (61.4%)	10 (17.5%)	
Total	33 (30.3%)	47 (43.1%)	29 (26.6%)		27 (24.8%)	59 (54.1%)	23 (21.1%)	

**Table 2:** The correlation between the right and left sides of the maximum graft length, width, and rugae. GPA: greater palatine artery.

	1	2	3	4	5	6	7	8
1. Left rugae	-	0.41 **	0.19 *	0.19 *	0.15	0.15	−0.63 **	−0.41 **
2. Right rugae		-	0.22 *	0.22 *	0.16	0.16	−0.37 **	−0.75 **
3. Left GPA			-	1.00 **	0.81 **	0.81 **	−0.07	−0.12
4. Left graft width				-	0.81 **	0.81 **	−0.07	−0.12
5. Right GPA					-	1.00 **	−0.08	−0.11
6. Right graft width						-	−0.08	−0.11
7. Left graft length							-	0.49 **
8. Right graft length								-

Note. ** *p* < 0.001, * *p* < 0.05.

## Data Availability

Data are available upon request.

## References

[1] Souza A.S.D., Mamatha H., Yothi N.D. (2012). Morphometric analysis of hard palate in south Indian skulls. Biomed. Res..

[2] Aleshkina O., Suetenkov D., Dydykin S., Vasil’ev Y., Paulsen F., Firsova I., Bikbaeva T., Polkovova I. (2021). Determination of sex dimorphisms of the thickness of the hard palate in adolescence using computed tomography: Pilot study. Ann. Anat.-Anat. Anz..

[3] Sarilita E., Soames R. (2016). Morphology of the Hard Palate: A Study of Dry Skulls and Review of the Literature. Rev. Argent. Anatomía Clínica.

[4] Araby Y.A., Alharbi A.S., Kolarkodi S.H., Almosyteer A.S. (2023). Morphometric analysis of the hard palate using cone beam computed tomography in a Saudi population. Saudi Dent. J..

[5] Hormdee D., Yamsuk T., Sutthiprapaporn P. (2020). Palatal Soft Tissue Thickness on Maxillary Posterior Teeth and Its Relation to Palatal Vault Angle Measured by Cone-Beam Computed Tomography. Int. J. Dent..

[6] Kim D.H., Won S.Y., Bae J.H., Jung U.W., Park D.S., Kim H.J., Hu K.S. (2014). Topography of the greater palatine artery and the palatal vault for various types of periodontal plastic surgery. Clin. Anat..

[7] Klosek S.K., Rungruang T. (2009). Anatomical study of the greater palatine artery and related structures of the palatal vault: Considerations for palate as the subepithelial connective tissue graft donor site. Surg. Radiol. Anat..

[8] Said K.N., Abu Khalid A.S., Farook F.F. (2021). Distal extension of palatal rugae as a limitation for donor soft tissue grafts in a Jordanian population: A cross-sectional study. BMC Oral Health.

[9] Reiser G.M., Bruno J.F., Mahan P.E., Larkin L.H. (1996). The subepithelial connective tissue graft palatal donor site: Anatomic considerations for surgeons. Int. J. Periodontics Restor. Dent..

[10] El Sergani A.M., Brandebura S., Padilla C., Butali A., Adeyemo W.L., Valencia-Ramírez C., Muñeton C.P.R., Moreno L.M., Buxó C.J., Neiswanger K. (2021). The Influence of Sex and Ancestry on Three-Dimensional Palate Shape. J. Craniofac. Surg..

[11] Al-Taai N., Persson M., Ransjö M., Levring Jäghagen E., Fors R., Westerlund A. (2022). Craniofacial changes from 13 to 62 years of age. Eur. J. Orthod..

[12] Kim D.W., Tempski J., Surma J., Ratusznik J., Raputa W., Świerczek I., Pękala J.R., Tomaszewska I.M. (2023). Anatomy of the greater palatine foramen and canal and their clinical significance in relation to the greater palatine artery: A systematic review and meta-analysis. Surg. Radiol. Anat..

[13] Gan N., Xiong Y., Jiao T. (2016). Accuracy of Intraoral Digital Impressions for Whole Upper Jaws, Including Full Dentitions and Palatal Soft Tissues. PLoS ONE.

[14] Rakosi T., Jonas I., Graber T.M. (1994). Color atlas of dental medicine, Orthodontic-Diagnosis. Am. J. Orthod. Dentofac. Orthop..

[15] Kang H., Huh S. (2021). Sample size determination and power analysis using the G*Power software. J. Educ. Eval. Health Prof..

[16] Beschiu L.M., Ardelean L.C., Tigmeanu C.V., Rusu L.-C. (2022). Cranial and Odontological methods for sex estimation—A scoping review. Medicina.

[17] Paul K.S., Feezell R., Hughes T., Brook A.H. (2023). Integrating genealogy and dental variation: Contributions to biological anthropology. Am. J. Biol. Anthropol..

[18] Avci S., Ergun T., Aydin E., Kansu L. (2015). Sex differences in adult craniofacial parameters. Surg. Radiol. Anat..

[19] Borusevičius R. (2023). Accuracy of Dental Implant Navigation Techniques and Biocompatibility of Materials Used for Immediate Prostheses. Ph.D. Thesis.

[20] Patil S.N., Naik S.B., Kamble S.D., Kokane V.B. (2015). To evaluate the accuracy of various dental parameters used for the gender determination in Nagpur District population. Indian J. Dent. Res..

[21] Gülcen B., Pelin İ., Özener E. (2021). The craniofacial indicators of aggression: A cross-sectional multiparametric anthropometry study. Folia Morphol..

[22] Ross J., Bowden M.R., Yu C., Diaz-Thomas A. (2023). Transition of young adults with metabolic bone diseases to adult care. Front. Endocrinol..

[23] Agarwal S.C. (2016). Bone morphologies and histories: Life course approaches in bioarchaeology. Am. J. Phys. Anthropol..

[24] Joganic J.L., Heuzé Y. (2019). Allometry and advancing age significantly structure craniofacial variation in adult female baboons. J. Anat..

[25] Cuozzo F.D., Espinosa M.M., da Silva K.T., de Barros Y.B., Bandeca M.C., Aranha A.M., Borges A.H., Volpato L.E. (2013). Cleft lip and palate in a Brazilian subpopulation. J. Int. Oral Health.

[26] Mohammad A., Koralakunte P.R. (2015). Gender identification and morphologic classification of tooth, arch and palatal forms in Saudi population. J. Pharm. Bioallied Sci..

[27] Fatima F., Fida M., Shaikh A. (2019). The association between palatal rugae pattern and dental malocclusion. Dent. Press J. Orthod..

[28] Mei Y.S., Syed Mohamed A.M.F., Marizan Nor M., Rosli T.I. (2023). Gender and age effects on dental and palatal arch dimensions among full siblings. J. Oral Sci..

[29] Gezer R., Deniz M., Uslu A.I. (2019). Morphological characteristics and individual differences of Palatal Rugae. J. Craniofacial Surg..

[30] Jain A., Chowdhary R. (2014). Palatal rugae and their role in forensic odontology. J. Investig. Clin. Dent..

[31] Shailaja A.M., Romana I.R.U., Narayanappa G., Smitha T., Gowda N.C., Vedavathi H.K. (2018). Assessment of palatal rugae pattern and its significance in orthodontics and forensic odontology. J. Oral Maxillofac. Pathol..

[32] Rengasamy Venugopalan S., Allareddy V. (2022). Craniofacial Growth and Development. Peterson’s Principles of Oral and Maxillofacial Surgery.

[33] Litsas G. (2013). Growth hormone therapy and craniofacial bones: A comprehensive review. Oral Dis..

[34] Santhosh Kumar S., Chacko R., Kaur A., Ibrahim G., Ye D. (2024). A systematic review of the use of intraoral scanning for human identification based on palatal morphology. Diagnostics.

[35] Puri K., Kumar A., Khatri M., Bansal M., Rehan M., Siddeshappa S.T. (2019). 44-year journey of palatal connective tissue graft harvest: A narrative review. J. Indian Soc. Periodontol..

[36] Labronici P.J., Tavares A.K., Canhoto E.C., Giordano V., Pires R.E.S., da Silva L.H.P., Mathias M.B., de Miranda Rosa I. (2017). Does the position of the scapula in relation to the glenopolar angle change the preferred treatment of extra-articular fractures?. Injury.

[37] Agbolade O., Nazri A., Yaakob R., Ghani A.A., Cheah Y.K. (2020). Morphometric approach to 3D soft-tissue craniofacial analysis and classification of ethnicity, sex, and age. PLoS ONE.

[38] Magnussen R.A., Lawrence J.T.R., West R.L., Toth A.P., Taylor D.C., Garrett W.E. (2012). Graft size and patient age are predictors of early revision after anterior cruciate ligament reconstruction with hamstring autograft. Arthrosc. J. Arthrosc. Relat. Surg..

[39] Cairo F., Cortellini P., Pilloni A., Nieri M., Cincinelli S., Amunni F., Pagavino G., Tonetti M.S. (2016). Clinical efficacy of coronally advanced flap with or without connective tissue graft for the treatment of multiple adjacent gingival recessions in the aesthetic area: A randomized controlled clinical trial. J. Clin. Periodontol..

[40] Perale G., Monjo M., Ramis J.M., Øvrebø Ø., Betge F., Lyngstadaas P., Haugen H.J. (2019). Biomimetic Biomolecules in next-generation Xeno-Hybrid bone graft material show enhanced in Vitro bone cells response. J. Clin. Med..

[41] Lloyd M.S., Buchanan E.P., Khechoyan D.Y. (2016). Review of quantitative outcome analysis of cranial morphology in craniosynostosis. J. Plast. Reconstr. Aesthetic Surg..

[42] Rutland J.W., Bellaire C.P., Yao A., Arrighi-Allisan A., Napoli J.G., Delman B.N., Taub P.J. (2021). The expanding role of geometric morphometrics in craniofacial surgery. J. Craniofacial Surg..

[43] Todorović A., Lisjak D., Lazic V., Špadijer-Gostović A. (2010). Possible errors during the optical impression procedure. Stomatol. Glas. Srb..

